# Discriminative Ability and Clinical Associations of Serum SIRT1, SIRT3, Apelin, and ELA in Patients with Diabetic Foot Infection

**DOI:** 10.3390/life16050804

**Published:** 2026-05-12

**Authors:** Revşa Evin Canpolat-Erkan, Recep Tekin, Aysun Ekinci, Fırat Aşır

**Affiliations:** 1 Department of Biochemistry, Faculty of Medicine, Dicle University, Diyarbakir 21280, Turkey; drevinerkan@gmail.com; 2 Department of Infectious Diseases and Clinical Microbiology, Faculty of Medicine, Dicle University, Diyarbakir 21280, Turkey; rectek21@hotmail.com; 3 Department of Histology and Embryology, Faculty of Medicine, Dicle University, Diyarbakir 21280, Turkey; firatasir@gmail.com

**Keywords:** diabetic foot infection, SIRT1, SIRT3, apelin, elabela

## Abstract

Background: Diabetic foot infection (DFI) is a serious complication of diabetes mellitus associated with chronic inflammation, impaired wound healing, endothelial dysfunction, and oxidative stress. Sirtuin (SIRT) signaling and the apelinergic system have been implicated in these processes. This study aimed to evaluate serum SIRT1, SIRT3, apelin, and elabela (ELA) levels in patients with DFI and to examine their cross-sectional associations with clinical indicators, inflammatory markers, osteomyelitis, and glycemic control. Methods: This cross-sectional study included 47 patients with DFI and 42 healthy controls. Serum biomarker levels were measured using enzyme-linked immunosorbent assay (ELISA). Clinical and laboratory data, including the infection component of the PEDIS classification, were recorded. Group comparisons, Spearman correlation analyses, receiver operating characteristic (ROC) curve analysis, and logistic regression were performed. Results: Patients with DFI exhibited higher inflammatory and glycemic markers and lower hemoglobin and lipid levels compared with controls (*p* < 0.05). Serum SIRT1, SIRT3, apelin, and ELA levels were significantly lower in the DFI group and showed inverse correlations with HbA1c, PEDIS stage, disease duration, osteomyelitis, and inflammatory markers. Among these biomarkers, SIRT1 showed the highest discriminative ability within this cohort (AUC = 0.820). In an exploratory multivariable model, age and SIRT1 were independently associated with the presence of DFI. Conclusions: Serum SIRT1, SIRT3, apelin, and ELA levels were lower in patients with DFI and were associated with clinical and biochemical indicators of disease burden. Among these biomarkers, SIRT1 demonstrated the strongest discriminative ability within this cohort. These findings suggest that sirtuin signaling and the apelinergic system may be relevant in the biological context of DFI; however, they should be interpreted cautiously. The observed differences may reflect not only DFI but also underlying diabetes, glycemic burden, age, and systemic inflammation. Further prospective studies including appropriate diabetic comparator groups are required to clarify the clinical relevance and potential utility of these biomarkers.

## 1. Introduction

Diabetic foot infection (DFI) is one of the most serious and debilitating complications of diabetes mellitus, contributing substantially to hospitalization, prolonged treatment, lower-extremity amputation, and mortality [1,2]. The development of DFI is multifactorial and involves peripheral neuropathy, impaired microvascular circulation, repetitive trauma, poor glycemic control, and defective wound healing [2,3]. Chronic hyperglycemia has been shown to promote endothelial dysfunction, oxidative stress, inflammatory activation, and impaired tissue perfusion, thereby creating a favorable environment for persistent infection and progressive tissue destruction [4]. Despite advances in wound care and antibiotic therapy, DFI remains a major clinical challenge due to delayed diagnosis, heterogeneous clinical presentation, and the limited availability of biomarkers that adequately reflect disease burden and healing potential [1].

Several inflammatory markers, including C-reactive protein (CRP), procalcitonin (PCT), erythrocyte sedimentation rate (ESR), and white blood cell (WBC) count, are widely used in the evaluation of DFI [5]. However, these markers primarily reflect systemic inflammation and infection burden and provide limited insight into underlying processes such as oxidative stress, mitochondrial dysfunction, endothelial impairment, and tissue repair. Given that diabetic wound healing is closely linked to metabolic dysregulation and microvascular dysfunction, there is growing interest in identifying novel biomarkers that may better capture the biological complexity of DFI [2,4].

Sirtuins (SIRTs) are a family of NAD+-dependent deacetylases involved in the regulation of cellular metabolism, oxidative stress, inflammation, mitochondrial homeostasis, and aging [6]. Among these, SIRT1 and SIRT3 have been extensively studied in the context of diabetic complications. Experimental evidence suggests that SIRT1 is involved in endothelial nitric oxide synthase activation, angiogenesis, macrophage polarization, and inflammatory regulation [4,7], whereas SIRT3 has been implicated in the maintenance of mitochondrial integrity, ATP production, reactive oxygen species detoxification, and cellular stress responses [8]. Reduced expression of SIRT1 and SIRT3 has been reported in association with diabetic nephropathy, retinopathy, cardiovascular disease, endothelial dysfunction, and impaired wound healing [8,9,10]. In experimental models, suppression of these pathways has been linked to delayed re-epithelialization, reduced fibroblast proliferation, persistent inflammation, and impaired angiogenesis in diabetic wounds [7,8].

The apelinergic system, comprising apelin, elabela (ELA), and the apelin receptor (APJ), has also been implicated in diabetic vascular complications. Apelin and ELA are endogenous peptides that contribute to endothelial homeostasis, vascular tone, tissue perfusion, angiogenesis, and oxidative stress regulation. Reduced levels of apelin and ELA have been reported in diabetic conditions associated with endothelial dysfunction, microvascular injury, and impaired tissue repair [11,12]. Experimental studies further suggest that apelin signaling may support wound healing through mechanisms such as enhanced blood flow, collagen synthesis, endothelial cell migration, and neovascularization [13,14]. In addition, emerging evidence indicates that the effects of the apelinergic system may be functionally linked to SIRT1- and SIRT3-related pathways [12], suggesting a potential interaction within a broader regulatory network involved in endothelial and mitochondrial homeostasis [4,12]. Although SIRT1, SIRT3, apelin, and ELA have been investigated individually in several diabetic complications, their combined relationship with DFI has not been well characterized. Data regarding their associations with clinical indicators, inflammatory markers, and disease burden in DFI remain limited.

Therefore, the present study aimed to evaluate serum SIRT1, SIRT3, apelin, and ELA levels in patients with DFI and to examine their cross-sectional associations with clinical indicators, inflammatory markers, and metabolic parameters. In addition, we assessed the discriminative ability of these biomarkers using receiver operating characteristic (ROC) analysis and explored their associations with the presence of DFI using logistic regression analysis. We hypothesized that reduced circulating levels of SIRT1, SIRT3, apelin, and ELA would be associated with increased disease burden and adverse clinical and biochemical profiles in patients with DFI.

## 2. Materials and Methods

### 2.1. Study Design and Participants

This single-center cross-sectional study was conducted between January 2024 and December 2024 at the infectious diseases and wound care units of a tertiary referral hospital. A total of 89 participants were enrolled, including 47 patients with DFI and 42 healthy controls. Patients with DFI were consecutively recruited from hospitalized individuals presenting with active diabetic foot wounds and clinical signs of infection. The diagnosis of DFI was based on the presence of diabetes mellitus together with local signs of infection, including purulent discharge, necrotic tissue, foul odor, sinus tract formation, erythema, increased local temperature, edema, or pain. Imaging findings were considered when clinically indicated. Magnetic resonance imaging (MRI) was used as the primary modality to evaluate deep tissue involvement and osteomyelitis. Osteomyelitis was defined by the presence of T1 hypointensity, T2/STIR hyperintensity, bone marrow edema, and cortical bone disruption on MRI. Plain radiography was used as a supportive tool to assess gross bone deformity or advanced osseous destruction.

The diagnosis of DFI was established based on standardized clinical criteria and was confirmed by an experienced infectious diseases specialist. Inclusion criteria for the DFI group were age > 18 years, a confirmed diagnosis of diabetes mellitus, elevated hemoglobin A1c (HbA1c) levels, and clinical findings consistent with diabetic foot infection. Exclusion criteria included autoimmune disease, congestive heart failure, end-stage renal disease, liver failure, malignancy, pregnancy, active systemic inflammatory disease unrelated to DFI, and the use of glucocorticoids or immunosuppressive agents. Healthy controls were selected from individuals without diabetes mellitus, active infection, chronic inflammatory disease, malignancy, or major systemic illness. Controls were younger than the DFI group, and this difference was considered in the interpretation of the results.

### 2.2. PEDIS Classification

The severity of diabetic foot infection was assessed using the PEDIS classification system proposed by the International Working Group on the Diabetic Foot [15]. The PEDIS system comprises five domains: perfusion, extent/size, depth/tissue loss, infection, and sensation. In the present study, only the infection component of the PEDIS classification was used. Accordingly, PEDIS stage 1 was defined as no infection, stage 2 as infection limited to the skin and subcutaneous tissue, stage 3 as diffuse cellulitis or deep tissue infection, and stage 4 as systemic inflammatory response syndrome associated with infection [16]. Based on these stages, patients were further categorized as having mild, moderate, or severe infection.

### 2.3. Biochemical Measurements

Blood samples were collected from patients with DFI within 24–48 h of hospital admission and prior to the initiation of parenteral antibiotics or intravenous fluid therapy. All patients presented with acute infection findings, including fever, purulent discharge, worsening wound pain, or local inflammatory changes. Venous blood samples were obtained after an overnight fast of approximately 12 h. Routine laboratory analyses included complete blood count, glucose, hemoglobin A1c (HbA1c), creatinine, lactate dehydrogenase (LDH), CRP, PCT, ESR, hemoglobin, total cholesterol, high-density lipoprotein cholesterol (HDL-C), low-density lipoprotein cholesterol (LDL-C), and triglycerides.

For biomarker analysis, 10 mL of venous blood was collected into standard biochemistry tubes, centrifuged at 1500× *g* for 20 min, and serum samples were separated. All samples were processed under standardized conditions, aliquoted to avoid repeated freeze–thaw cycles, and stored at −80 °C until analysis [17]. Serum SIRT1, SIRT3, apelin, and ELA concentrations were measured using commercially available enzyme-linked immunosorbent assay (ELISA) kits (Human SIRT1 ELISA, Human SIRT3 ELISA, Human apelin ELISA, Human ELA ELISA; Sunred Biological Technology, Shanghai, China) according to the manufacturers’ instructions. All measurements were performed in duplicate to ensure analytical reliability. Clinical chemistry tests and CRP were measured spectrophotometrically using a Beckman Coulter AU-5800 autoanalyzer (Beckman Coulter, Brea, CA, USA). Hemogram measurements were performed using a SYSMEX XN-1000 analyzer (Sysmex, Kobe, Japan). HbA1c levels were measured using a Variant II Turbo HbA1c analyzer (Bio-Rad Laboratories, Hercules, CA, USA) by high-performance liquid chromatography (HPLC). PCT levels were determined using an electrochemiluminescence method with a Beckman Coulter DXI 800 analyzer (Beckman Coulter, Brea, CA, USA).

### 2.4. Outcomes

The primary outcome of the study was the difference in serum SIRT1, SIRT3, apelin, and ELA levels between patients with DFI and healthy controls. Secondary outcomes included the cross-sectional associations between these biomarkers and clinical and laboratory parameters, including PEDIS stage, osteomyelitis, glycemic control, inflammatory markers, and disease duration.

### 2.5. Statistical Analysis

Statistical analyses were performed using IBM SPSS Statistics software (Version 25.0; IBM Corp., Armonk, NY, USA). Continuous variables were tested for normality using the Shapiro–Wilk test. Normally distributed variables were presented as mean ± standard deviation, whereas non-normally distributed variables were expressed as median (interquartile range). Categorical variables were presented as numbers and percentages. Comparisons between patients with DFI and healthy controls were performed using the independent samples *t*-test for normally distributed variables and the Mann–Whitney U test for non-normally distributed variables. Comparisons among PEDIS subgroups were performed using the Kruskal–Wallis test, followed by Dunn–Bonferroni post hoc analysis when appropriate. Categorical variables were compared using the chi-square test or Fisher’s exact test.

Correlations between biomarker levels and clinical and laboratory variables were assessed using Spearman’s rank correlation analysis due to non-normal distributions. Receiver operating characteristic (ROC) curve analysis was performed to evaluate their discriminative ability between patients with DFI and healthy controls. The area under the curve (AUC), 95% confidence intervals (CIs), optimal cut-off values, sensitivity, and specificity were calculated.

Univariate logistic regression analysis was performed to explore variables associated with the presence of DFI. Variables with clinical relevance and significant univariate associations were included in a multivariable logistic regression model. To minimize multicollinearity, only SIRT1 was included among the biomarker variables in the multivariable model due to the strong intercorrelations observed among SIRT1, SIRT3, apelin, and ELA. Odds ratios (ORs) and 95% CIs were reported. Given the relatively small sample size, the multivariable analysis was considered exploratory. Variables included in the multivariable model were selected based on clinical relevance and univariate significance. A two-sided *p*-value < 0.05 was considered statistically significant.

### 2.6. Ethical Approval

The study was conducted in accordance with the Declaration of Helsinki and was approved by the Dicle University Faculty of Medicine Ethics Committee (Approval date: 17 January 2023, approval number: 289). Written informed consent was obtained from all participants prior to enrollment.

## 3. Results

### 3.1. Baseline Demographic, Clinical, Laboratory, and Biomarker Characteristics of the Study Population

The demographic and clinical characteristics of the study population are presented in Table 1. A total of 89 participants were included, comprising 47 patients with DFI and 42 healthy controls. Patients with DFI were older than controls (59.9 ± 8.5 vs. 44.8 ± 7.4 years, *p* < 0.001), whereas sex distribution and body mass index (BMI) were similar between the groups (*p* > 0.05). Compared with controls, patients with DFI had higher WBC count, glucose, creatinine, LDH, CRP, PCT, ESR, and HbA1c levels (all *p* < 0.05). Hemoglobin, total cholesterol, HDL-C, and LDL-C levels were lower in the DFI group than in controls (all *p* < 0.05), whereas triglyceride levels did not differ significantly between the groups (*p* = 0.433).

Serum SIRT1, SIRT3, apelin, and ELA levels were lower in patients with DFI compared with controls. Median SIRT1 levels were 12.6 (4.5–18.7) ng/mL in controls and 3.2 (2.9–6.3) ng/mL in patients with DFI (*p* < 0.001). Median SIRT3 levels were 33.2 (9.1–69.0) ng/mL in controls and 5.9 (5.1–12.5) ng/mL in the DFI group (*p* < 0.001). Median apelin levels were 72.9 (32.3–112.0) pg/mL in controls and 27.7 (23.4–43.1) pg/mL in patients with DFI (*p* = 0.001). Median ELA levels were 832.5 (441.7–1292.6) ng/mL in controls and 303.2 (248.6–510.4) ng/mL in the DFI group (*p* = 0.001).

### 3.2. Clinical and Biomarker Differences According to PEDIS Stage in Patients with Diabetic Foot Infection

Clinical and laboratory parameters according to PEDIS stage are presented in Table 2. Among the evaluated variables, CRP levels and the frequency of osteomyelitis differed across PEDIS categories. CRP levels were higher in patients with advanced PEDIS stages (*p* = 0.006). Osteomyelitis was observed predominantly in patients in PEDIS stages 3 and 4 (*p* < 0.001). Glucose, HbA1c, ESR, and PCT levels showed higher median values in higher PEDIS stages; however, these differences were not statistically significant (all *p* > 0.05). Similarly, SIRT1, SIRT3, apelin, and ELA levels differed across PEDIS categories, but these differences did not reach statistical significance (all *p* > 0.05). Patients classified as PEDIS stage 1 were included based on clinical suspicion of infection at presentation but were categorized as stage 1 according to classification criteria.

### 3.3. Correlations Between Serum Biomarkers and Clinical/Laboratory Parameters

Spearman correlation coefficients between serum SIRT1, SIRT3, apelin, ELA, and clinical and laboratory parameters in the overall study population are presented in Table 3. Serum SIRT1, SIRT3, apelin, and ELA levels showed negative correlations with diabetes duration, DFI duration, HbA1c, PEDIS stage, osteomyelitis, and PCT levels (all *p* < 0.05). SIRT1 and SIRT3 levels were also negatively correlated with age, glucose, ESR, and LDH (all *p* < 0.05). Among the evaluated biomarkers, SIRT1 showed the strongest negative correlation with PEDIS stage (r = −0.546, *p* < 0.001), followed by SIRT3 (r = −0.437, *p* < 0.001), ELA (r = −0.388, *p* < 0.001), and apelin (r = −0.365, *p* < 0.001). Positive correlations were observed between biomarker levels and lipid parameters, particularly HDL-C. Strong positive correlations were also observed among SIRT1, SIRT3, apelin, and ELA (r = 0.782–0.886, all *p* < 0.001).

### 3.4. Discriminative Ability of Serum Biomarkers Between Patients with DFI and Healthy Controls

ROC curve analysis results for serum biomarkers and inflammatory markers are presented in Table 4. All evaluated biomarkers showed varying levels of discriminative ability within this cohort. Among the investigated biomarkers, SIRT1 had the highest AUC = 0.820, followed by SIRT3 (AUC = 0.750), ELA (AUC = 0.713), and apelin (AUC = 0.704). Conventional inflammatory markers showed higher AUC values, with PCT (AUC = 0.945) and CRP (AUC = 0.869) demonstrating the highest values among all variables. SIRT1 showed a higher AUC compared with the other evaluated biomarkers.

### 3.5. ROC Curve Analysis of SIRT1, SIRT3, Apelin, and ELA

**Figure 1:**
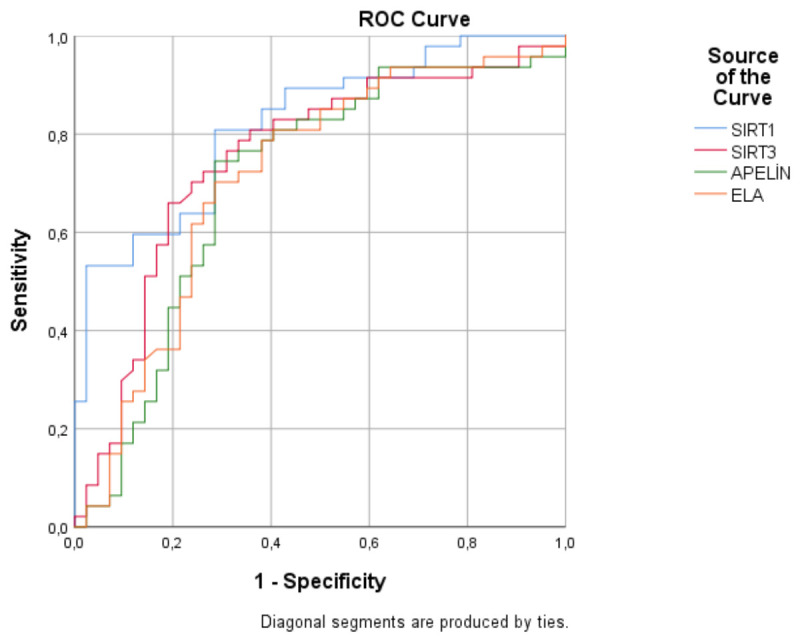
ROC curves of serum SIRT1, SIRT3, apelin, and ELA for differentiating patients with DFI from healthy controls. The AUC values were 0.820 for SIRT1, 0.750 for SIRT3, 0.713 for ELA, and 0.704 for apelin.

ROC curve analysis showed that SIRT1 had the highest AUC = 0.820 among the evaluated biomarkers. The AUC values for SIRT3, ELA, and apelin were 0.750, 0.713, and 0.704, respectively. ROC curve analysis findings are illustrated in Figure 1.

### 3.6. Logistic Regression Analysis of Independent Predictors of Diabetic Foot Infection

Univariate and multivariable logistic regression analyses are presented in Table 5. In univariate analysis, age, CRP, PCT, SIRT1, SIRT3, apelin, and ELA were associated with the presence of DFI (all *p* < 0.05). Older age, higher CRP, and higher PCT levels were associated with increased odds of DFI, whereas higher SIRT1, SIRT3, apelin, and ELA levels were associated with lower odds. In the multivariable model including age, CRP, and SIRT1, age and SIRT1 remained independently associated with the presence of DFI in an exploratory model. Increasing age was associated with higher odds of DFI (OR = 1.40, 95% CI: 1.13–1.74, *p* = 0.002), whereas higher SIRT1 levels were associated with lower odds (OR = 0.81, 95% CI: 0.67–0.97, *p* = 0.021). CRP was not statistically significant after adjustment. Given the relatively small sample size, the multivariable analysis should be interpreted as exploratory.

## 4. Discussion

The present study showed that serum SIRT1, SIRT3, apelin, and ELA levels were lower in patients with DFI compared with healthy controls, whereas conventional inflammatory markers such as CRP, PCT, ESR, and LDH were higher in the DFI group. These findings suggest that DFI may be associated with alterations in processes related to oxidative stress, mitochondrial function, endothelial regulation, and tissue repair. The use of healthy controls in this study reflects a proof-of-concept design aimed at identifying a biomarker signal rather than establishing disease specificity. Therefore, the observed discriminative ability should not be interpreted as disease-specific diagnostic accuracy. While this approach allows for the detection of measurable differences, it does not permit differentiation between DFI-specific effects and those related to diabetes, metabolic dysregulation, age, or systemic inflammation. Among the evaluated biomarkers, SIRT1 showed the strongest inverse correlations with PEDIS stage, HbA1c, glucose, inflammatory parameters, and osteomyelitis. In ROC analysis, SIRT1 had the highest AUC among the investigated biomarkers, and in multivariable analysis it remained independently associated with the presence of DFI. However, these findings should be interpreted with caution, as the regression model may be affected by limited sample size and potential quasi-separation. Therefore, the multivariable results should be considered exploratory rather than definitive. In addition, osteomyelitis, which was more frequently observed in advanced PEDIS stages, may have contributed to the observed biomarker alterations, and its independent effect cannot be fully distinguished. Given the significant age difference between the groups, residual confounding cannot be excluded, and part of the observed biomarker differences may be attributable to age-related changes rather than DFI itself.

SIRT1 is an NAD+-dependent deacetylase that has been implicated in the regulation of oxidative stress, inflammatory signaling, mitochondrial homeostasis, angiogenesis, and apoptosis [18]. Experimental studies suggest that reduced SIRT1 activity may contribute to impaired wound healing through increased reactive oxygen species production, endothelial dysfunction, persistent inflammatory activation, and reduced fibroblast proliferation [19,20]. In diabetic wound models, suppression of SIRT1 has been associated with delayed re-epithelialization and impaired neovascularization [19,21]. In addition, SIRT1 has been linked to endothelial nitric oxide synthase activation, macrophage polarization, and maintenance of endothelial homeostasis, and reduced expression has been associated with a shift toward a pro-inflammatory phenotype and impaired tissue repair [22,23]. Clinical studies have also reported associations between low SIRT1 expression and adverse vascular outcomes in diabetic populations [23,24]. Taken together, the reduced SIRT1 levels observed in the present study may be associated with both increased inflammatory burden and impaired regenerative processes in patients with DFI. However, these interpretations are based on previously reported experimental findings and should not be considered direct mechanistic evidence in the context of the present study.

SIRT3, another member of the sirtuin family, also showed lower levels in patients with DFI and was negatively correlated with PEDIS stage, HbA1c, PCT, and disease duration. SIRT3 is primarily localized in mitochondria and has been implicated in the regulation of mitochondrial oxidative metabolism and antioxidant defense. Reduced SIRT3 expression has been associated with mitochondrial dysfunction, increased reactive oxygen species accumulation, impaired ATP production, and enhanced inflammatory signaling in diabetic tissues [25,26,27]. Experimental studies further suggest that SIRT3 may contribute to mitochondrial stability, energy production, and modulation of inflammatory responses [27,28]. In this context, lower SIRT3 levels observed in patients with DFI may be associated with impaired cellular repair capacity and persistent tissue injury. However, these interpretations are based on experimental evidence and should not be considered direct mechanistic conclusions. In addition, the higher age and poorer glycemic control (HbA1c) observed in the DFI group may have influenced biomarker levels, as both aging and hyperglycemia are known to affect oxidative stress, mitochondrial function, and sirtuin activity. Therefore, the observed reductions in SIRT1, SIRT3, apelin, and ELA levels may not be exclusively attributable to DFI but may also reflect underlying metabolic and age-related factors. The lack of full adjustment for these potential confounders represents an important limitation and should be considered when interpreting the findings. In addition, the extremely large odds ratio observed for PCT reflects model instability and quasi-separation, and therefore should not be interpreted quantitatively.

Apelin and ELA, which are endogenous ligands of the APJ receptor system [29], were also lower in patients with DFI. The apelinergic system has been implicated in the regulation of vascular integrity, angiogenesis, tissue perfusion, and endothelial function. Previous studies suggest that apelin may support wound healing through effects on angiogenesis, collagen synthesis, and keratinocyte migration [30,31]. Similarly, ELA has been reported to exert protective effects on endothelial cells and microvascular function [32,33]. In diabetic conditions, reduced apelin expression has been observed in ischemic tissues, and lower ELA levels have been associated with microvascular complications and endothelial dysfunction [5,32,34]. In this context, decreased apelin and ELA levels observed in DFI may be associated with impaired tissue perfusion, altered angiogenic responses, and microvascular dysfunction. However, these interpretations are based on previously reported experimental findings and should not be considered direct mechanistic evidence. Experimental studies further suggest potential interactions between the apelinergic system and SIRT1- and SIRT3-related pathways, including AMPK-mediated signaling [35,36,37]. Accordingly, the simultaneous reduction in SIRT1, SIRT3, apelin, and ELA observed in the present study may reflect related biological processes. This may partly explain the strong positive correlations observed among these biomarkers, although such relationships cannot be interpreted as evidence of a unified regulatory pathway within the scope of the present study.

Although biomarker levels varied across PEDIS stages, subgroup comparisons did not show statistically significant differences. In contrast, significant inverse correlations were observed between biomarker levels and PEDIS stage. This discrepancy may be related to methodological differences, as correlation analysis can be more sensitive in detecting monotonic trends across ordinal categories than group-based comparisons. In addition, the relatively small number of patients in advanced PEDIS stages may have limited the statistical power of subgroup analyses. These limitations further support interpreting the multivariable analysis as exploratory rather than confirmatory.

ROC analysis indicated that the evaluated biomarkers differ in their ability to discriminate between patients with DFI and healthy controls, with SIRT1 showing the highest AUC among the investigated biomarkers, followed by SIRT3, ELA, and apelin. Conventional inflammatory markers, particularly CRP and PCT, showed higher AUC values.

While these findings suggest that SIRT1 and related biomarkers may provide additional information beyond conventional inflammatory markers, they should be interpreted with caution. These biomarkers may reflect biological processes such as oxidative stress, mitochondrial function, and endothelial regulation that are not directly captured by routine inflammatory parameters. However, given the cross-sectional design and the use of healthy controls instead of diabetic comparator groups, no conclusions can be drawn regarding their disease-specific clinical utility in practice. Accordingly, these biomarkers may be considered as complementary indicators reflecting underlying biological processes rather than replacements for established clinical markers. Further studies are required to determine whether their combined use with conventional markers improves discriminative ability or provides additional clinical insight.

It should be emphasized that the present study does not provide direct mechanistic evidence. The observed associations between serum biomarkers and clinical parameters should be interpreted within the context of biological plausibility based on previous experimental and preclinical studies, rather than as confirmation of underlying molecular mechanisms. Accordingly, the potential roles of SIRT1, SIRT3, and the apelinergic system in DFI pathophysiology should be considered hypothesis-generating and require further validation in mechanistic and longitudinal studies.

Several limitations of the present study should be acknowledged. First, the study had a single-center cross-sectional design with a relatively small sample size, which limits generalizability and precludes causal or prognostic inference. Second, serial biomarker measurements were not available; therefore, temporal changes during treatment and wound healing could not be evaluated. Third, tissue-level expression analyses, mechanistic experiments, and molecular pathway studies were not performed. Another important limitation is the absence of a comparator group consisting of diabetic patients without DFI. Therefore, it is not possible to determine whether the observed reductions in SIRT1, SIRT3, apelin, and ELA levels are specific to DFI or reflect underlying diabetes-related metabolic dysregulation, systemic inflammation, aging, or other comorbid conditions. In addition, the higher age and poorer glycemic control observed in the DFI group may have contributed to the observed differences. The relatively small sample size and the limited number of patients in advanced PEDIS stages may have reduced the statistical power of subgroup analyses. Furthermore, information on additional clinical covariates, including diabetes type, treatment regimens, vascular status, microbiological findings, prior antibiotic exposure, smoking status, peripheral arterial disease, and medication use, was not consistently available. Despite these limitations, the findings of the present study should be considered exploratory and hypothesis-generating. Further studies with larger sample sizes, appropriate diabetic comparator groups, and longitudinal designs are required to clarify the clinical relevance and potential role of these biomarkers in DFI.

## 5. Conclusions

In this cross-sectional study, serum SIRT1, SIRT3, apelin, and ELA levels were lower in patients with DFI compared with healthy controls and were associated with clinical and biochemical indicators of disease burden. Among these biomarkers, SIRT1 showed the highest discriminative ability within this cohort.

These findings should be interpreted as exploratory and hypothesis-generating. The observed associations do not establish causality or disease-specific clinical utility, and the clinical relevance of these biomarkers beyond conventional inflammatory markers remains to be determined. Further large-scale, prospective studies including appropriate diabetic comparator groups are required to clarify their potential role and determine disease specificity.

## Figures and Tables

**Table 1:** Comparison of demographic, clinical, laboratory, and biomarker characteristics between healthy controls and patients with diabetic foot infection.

Variable	Control	DFI	*p*
Age (years)	44.8 ± 7.4	59.9 ± 8.5	<0.001
BMI (kg/m^2^)	25.0 (22.0–28.0)	25.0 (23.0–27.5)	0.596
WBC count (10^3^/µL)	7700 (6200–9000)	8900 (7450–11,000)	0.007
Hb (g/dL)	14.0 (13.0–15.0)	11.0 (10.0–13.0)	<0.001
Glucose (mg/dL)	84 (75–93)	166 (125–230.5)	<0.001
Creatinine (mg/dL)	0.7 (0.6–0.9)	0.9 (0.6–1.1)	0.013
LDH (U/L)	184 (157–207)	209 (188–246)	0.001
ESR (mm/h)	5 (3–11)	49 (24–68)	<0.001
CRP (mg/L)	1.2 (0.4–2.9)	31.0 (6.4–93.5)	<0.001
PCT (µg/L)	0.02 (0.01–0.02)	0.10 (0.05–0.20)	<0.001
Total cholesterol (mg/dL)	216.3 ± 44.2	161.2 ± 38.9	<0.001
Triglycerides (mg/dL)	148 (92–211)	161 (114–219)	0.433
HDL-C (mg/dL)	55.4 ± 9.9	35.2 ± 11.4	<0.001
LDL-C (mg/dL)	126.8 ± 36.9	94.0 ± 31.5	<0.001
HbA1c (%)	5.4 ± 0.4	9.5 ± 2.1	<0.001
SIRT1 (ng/mL)	12.6 (4.5–18.7)	3.2 (2.9–6.3)	<0.001
SIRT3 (ng/mL)	33.2 (9.1–69.0)	5.9 (5.1–12.5)	<0.001
Apelin (pg/mL)	72.9 (32.3–112.0)	27.7 (23.4–43.1)	0.001
ELA (ng/mL)	832.5 (441.7–1292.6)	303.2 (248.6–510.4)	0.001

Data are presented as mean ± SD or median (IQR). Comparisons were performed using the *t*-test or Mann–Whitney U test, and categorical variables were compared using the chi-square test. Abbreviations: BMI, body mass index; CRP, C-reactive protein; DFI, diabetic foot infection; ESR, erythrocyte sedimentation rate; ELA, elabela; Hb, hemoglobin; HbA1c, hemoglobin A1c; HDL-C, high-density lipoprotein cholesterol; LDH, lactate dehydrogenase; LDL-C, low-density lipoprotein cholesterol; PCT, procalcitonin; SIRT, sirtuin; WBC, white blood cell.

**Table 2:** Comparison of clinical, laboratory, and biomarker parameters according to PEDIS stage in patients with diabetic foot infection.

Variable	PEDIS 1 (n = 10)	PEDIS 2 (n = 14)	PEDIS 3 (n = 17)	PEDIS 4 (n = 6)	*p*
Duration of diabetes (years)	10.0 (5.0–15.0)	13.5 (7.8–20.0)	15.0 (12.0–18.0)	15.0 (15.0–15.0)	0.395
Duration of ulcer (days)	52.5 (30.0–82.5)	17.5 (14.0–41.3)	15.0 (10.0–20.0)	14.5 (8.8–37.5)	0.364
Osteomyelitis, n (%)	0 (0)	0 (0)	16 (94.1)	5 (83.3)	<0.001
Glucose (mg/dL)	144.0 (114.3–177.5)	153.0 (102.5–217.8)	192.0 (164.0–310.0)	172.5 (160.5–203.3)	0.169
HbA1c (%)	8.35 (7.50–10.30)	8.85 (7.63–10.45)	10.00 (8.70–11.40)	9.65 (8.18–11.65)	0.305
CRP (mg/L)	6.7 (1.6–14.0)	33.5 (5.8–74.8)	64.0 (9.0–128.0)	197.5 (60.5–331.5)	0.006
PCT (µg/L)	0.10 (0.06–0.18)	0.10 (0.07–0.20)	0.10 (0.10–0.13)	0.15 (0.10–0.20)	0.521
ESR (mm/h)	41.0 (15.0–47.5)	47.5 (8.5–61.8)	61.0 (39.0–84.0)	66.0 (57.5–70.0)	0.077
SIRT1 (ng/mL)	4.21 (3.31–5.17)	3.24 (2.94–9.72)	3.01 (2.76–3.53)	4.03 (2.89–6.53)	0.566
SIRT3 (ng/mL)	8.54 (5.64–12.03)	6.14 (5.28–14.28)	5.60 (4.93–7.48)	6.64 (5.29–11.33)	0.671
Apelin (pg/mL)	31.78 (27.11–38.85)	34.92 (24.34–70.54)	25.81 (22.48–34.51)	27.51 (26.45–33.28)	0.593
ELA (ng/mL)	356.15 (267.57–805.02)	329.61 (272.71–512.30)	290.84 (227.12–356.50)	300.28 (282.39–491.51)	0.383

Data are presented as median (IQR) or n (%). Comparisons among PEDIS groups were performed using the Kruskal–Wallis test for continuous variables and the chi-square test for categorical variables. Abbreviations: CRP, C-reactive protein; ESR, erythrocyte sedimentation rate; ELA, elabela; HbA1c, hemoglobin A1c; PCT, procalcitonin; PEDIS, Perfusion, Extent/Size, Depth/Tissue Loss, Infection, and Sensation; SIRT, sirtuin.

**Table 3:** Spearman correlation analysis between serum biomarkers and clinical/laboratory parameters.

Variable	SIRT1	SIRT3	Apelin	ELA
Age	−0.307	−0.243	−0.129	−0.154
Duration of diabetes	−0.415	−0.239	−0.219	−0.347
Duration of DFI	−0.289	−0.246	−0.264	−0.264
Glucose	−0.356	−0.311	−0.189	−0.216
HbA1c	−0.532	−0.468	−0.362	−0.392
PEDIS stage	−0.546	−0.437	−0.365	−0.388
Osteomyelitis	−0.294	−0.238	−0.287	−0.304
ESR	−0.276	−0.210	−0.191	−0.135
PCT	−0.421	−0.304	−0.238	−0.248
LDH	−0.247	−0.229	−0.210	−0.176
Total cholesterol	0.335	0.242	0.186	0.220
HDL-C	0.464	0.367	0.287	0.309
LDL-C	0.241	0.163	0.103	0.123
SIRT3	0.864	—	—	—
Apelin	0.782	0.861	—	—
ELA	0.833	0.886	0.861	—

Correlation coefficients were calculated using Spearman’s rank correlation analysis. Abbreviations: DFI, diabetic foot infection; ELA, elabela; ESR, erythrocyte sedimentation rate; HbA1c, hemoglobin A1c; HDL-C, high-density lipoprotein cholesterol; LDH, lactate dehydrogenase; LDL-C, low-density lipoprotein cholesterol; PEDIS, Perfusion, Extent/Size, Depth/Tissue Loss, Infection, and Sensation; PCT, procalcitonin; SIRT, sirtuin. All reported correlations were statistically significant (*p* < 0.05) unless otherwise specified. “—” indicates variables not included in the multivariable analysis.

**Table 4:** Discriminative ability of serum biomarkers for differentiating patients with diabetic foot infection from healthy controls.

Variable	AUC (95% CI)	Cut-Off	Sensitivity (%)	Specificity (%)	*p*
SIRT1	0.820 (0.731–0.909)	≤6.5 ng/mL	78.7	76.2	<0.001
SIRT3	0.750 (0.647–0.853)	≤8.9 ng/mL	72.3	69.0	<0.001
Apelin	0.704 (0.592–0.815)	≤35.0 pg/mL	68.1	66.7	0.002
ELA	0.713 (0.602–0.824)	≤420 ng/mL	70.2	69.0	0.001
CRP	0.869 (0.792–0.946)	>12.5 mg/L	83.0	81.0	<0.001
PCT	0.945 (0.897–0.993)	>0.05 µg/L	89.4	88.1	<0.001

Cut-off values were determined based on ROC analysis. Sensitivity and specificity are presented as percentages. Abbreviations: AUC, area under the curve; CI, confidence interval; CRP, C-reactive protein; ELA, elabela; PCT, procalcitonin; SIRT, sirtuin.

**Table 5:** Univariate and multivariate logistic regression analysis for predictors of DFI.

Variable	Univariate OR (95% CI)	*p*	Multivariate OR (95% CI)	*p*
Age	1.40 (1.21–1.61)	<0.001	1.40 (1.13–1.74)	0.002
HbA1c	Not stable due to quasi-separation	—	—	—
CRP	1.18 (1.05–1.32)	0.007	1.09 (0.98–1.21)	0.129
PCT	6.17 × 10^31^ (1.26 × 10^19^–3.01 × 10^44^)	<0.001	—	—
SIRT1	0.86 (0.80–0.92)	<0.001	0.81 (0.67–0.97)	0.021
SIRT3	0.99 (0.98–0.997)	0.008	—	—
Apelin	0.98 (0.98–0.99)	0.002	—	—
ELA	0.999 (0.998–0.999)	0.001	—	—

Variables with significant univariate associations and clinical relevance were included in the multivariable model. “—” indicates variables not included in the multivariable analysis. HbA1c could not be reliably estimated due to quasi-separation. Abbreviations: CI, confidence interval; CRP, C-reactive protein; ELA, elabela; HbA1c, hemoglobin A1c; OR, odds ratio; PCT, procalcitonin; SIRT, sirtuin.

## Data Availability

All data generated or analyzed during this study are included in this article. Further inquiries can be directed to the corresponding author.
